# MXD3 as an onco-immunological biomarker encompassing the tumor microenvironment, disease staging, prognoses, and therapeutic responses in multiple cancer types

**DOI:** 10.1016/j.csbj.2021.08.047

**Published:** 2021-09-02

**Authors:** Szu-Yuan Wu, Kuan-Chou Lin, Bashir Lawal, Alexander T.H Wu, Ching-Zong Wu

**Affiliations:** a Department of Food Nutrition and Health Biotechnology, College of Medical and Health Science, Asia University, Taichung, Taiwan; b Big Data Center, Lo-Hsu Medical Foundation, Lotung Poh-Ai Hospital, Yilan, Taiwan; c Division of Radiation Oncology, Lo-Hsu Medical Foundation, Lotung Poh-Ai Hospital, Yilan, Taiwan; d Department of Healthcare Administration, College of Medical and Health Science, Asia University, Taichung, Taiwan; e Graduate Institute of Business Administration, Fu Jen Catholic University, New Taipei City, Taiwan; f Centers for Regional Anesthesia and Pain Medicine, Wan Fang Hospital, Taipei Medical University, Taipei, Taiwan; g Cancer Center, Lo-Hsu Medical Foundation, Lotung Poh-Ai Hospital, Yilan, Taiwan; h School of Dentistry, College of Oral Medicine, Taipei Medical University, Taipei, Taiwan; i Division of Oral and Maxillofacial Surgery, Department of Dentistry, Wan Fang Hospital, Taipei Medical University, Taipei, Taiwan; j Graduate Institute for Cancer Biology & Drug Discovery, College of Medical Science and Technology, Taipei Medical University, Taipei, Taiwan; k PhD Program for Cancer Molecular Biology and Drug Discovery, College of Medical Science and Technology, Taipei Medical University and Academia Sinica, Taipei, Taiwan; l The PhD Program of Translational Medicine, College of Medical Science and Technology, Taipei Medical University, Taipei, Taiwan; m Clinical Research Center, Taipei Medical University Hospital, Taipei Medical University, Taipei, Taiwan; n Graduate Institute of Medical Sciences, National Defense Medical Center, Taipei, Taiwan; o Taipei Heart Institute (THI), Taipei Medical University, Taipei, Taiwan; p Department of Dentistry, Taipei Medical University Hospital, Taipei, Taiwan; q Department of Dentistry, Lotung Poh-Ai hospital, Yilan, Taiwan

**Keywords:** MXD3, Immuno-oncology, Immunotherapy, Chemotherapy, Gene expression profiling, Immune-cell infiltration, T-cell exclusion, Genetic and epigenetic alterations

## Abstract

MAX dimerization (MXD) protein 3 (MXD3) is a member of the MXD family of basic-helix-loop-helix-leucine-zipper (bHLHZ) transcription factors that plays pivotal roles in cell cycle progression and cell proliferation. However, there is insufficient scientific evidence on the pathogenic roles of MXD3 in various cancers and whether MXD3 plays a role in the immuno-oncology context of the tumor microenvironment, pathogenesis, prognosis, and therapeutic response of different tumors through certain common molecular mechanisms; thus, we saw a need to conduct the present *in silico* pan-cancer study. Using various computational tools, we interrogated the role of MXD3 in tumor immune infiltration, immune evasion, tumor progression, therapy response, and prognosis of cohorts from various cancer types. Our results indicated that MXD3 was aberrantly expressed in almost all The Cancer Genome Atlas (TCGA) cancer types and subtypes and was associated with the tumor stage, metastasis, and worse prognoses of various cohorts. Our results also suggested that MXD3 is associated with tumor immune evasion via different mechanisms involving T-cell exclusion in different cancer types and by tumor infiltration of immune cells in thymoma (THYM), liver hepatocellular carcinoma (LIHC), and head and neck squamous cell carcinoma (HNSC). Methylation of MXD3 was inversely associated with messenger (m)RNA expression levels and mediated dysfunctional T-cell phenotypes and worse prognoses of cohorts from different cancer types. Finally, we found that genetic alterations and oncogenic features of MXD3 were concomitantly associated with deregulation of the *DBN1*, *RAB24*, *SLC34A1*, *PRELID1*, *LMAN2*, *F12*, *GRK6*, *RGS14*, *PRR7*, and *PFN3* genes and were connected to phospholipid transport and ion homeostasis. Our results also suggested that MXD3 expression is associated with immune or chemotherapeutic outcomes in various cancers. In addition, higher MXD3 expression levels were associated with decreased sensitivity of cancer cell lines to several mitogen-activated protein kinase kinase (MEK) inhibitors but led to increased activities of other kinase inhibitors, including Akt inhibitors. Interestingly, MXD3 exhibited higher predictive power for response outcomes and overall survival of immune checkpoint blockade sub-cohorts than three of seven standardized biomarkers. Altogether, our study strongly suggests that MXD3 is an immune-oncogenic molecule and could serve as a biomarker for cancer detection, prognosis, therapeutic design, and follow-up.

## Graphical abstract

**Figure:**
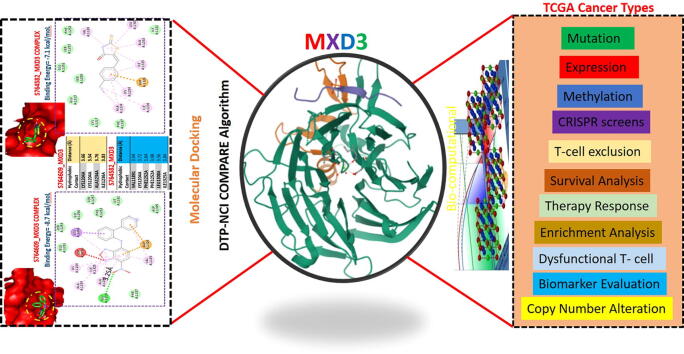

## 1 Introduction

We now know that the initiation and progression of cancer are multistage processes that result from the accumulation of both genetic and epigenetic alterations of the genome [1]. However, contrary to our initial view that genetic alterations and epigenetic modifications are two distinct mechanisms of carcinogenesis [2], accumulating evidence has demonstrated that genetic alterations can disrupt several epigenetic patterns, while epigenetic modifications can drive genomic instability and mutagenesis [3], [4], [5], suggesting that there is a crosstalk between genetic and epigenetic alterations during carcinogenesis [6].

The tumor microenvironment (TME) is a diverse ecological niche consisting of heterogeneous clones of tumor cells and normal cells, including fibroblasts, the vasculature, and an extensive pool of immune cells and immunosuppressive cells [7], [8]. This complexity results in an interplay of various cellular signaling systems, where tumor cells infiltrate immune cells and render them dysfunctional and thus unable to mount any antitumor immune actions via a process known as T-cell anergy [9], [10], [11]; instead, the tumor immune cell component is now established to mediate cancer progression and therapeutic responses [12], [13], [14], [15]. In addition, the immunosuppressive cell component of the TME, including M2 subtypes of tumor-associated macrophages (TAMs), cancer-associated fibroblasts (CAFs), regulatory T cells (Tregs), and myeloid-derived suppressor cells (MDSCs), can inhibit the production, activity, and infiltration of cytotoxic T cells, and promote tumor immune evasion, tumor growth, metastasis, and therapeutic resistance [16], [17].

Unfortunately, it is experimentally laborious and challenging to systematically profile these distinct immune and immunosuppressive cell types from heterogeneous tumor samples in order to identify and characterize potential therapeutic targets and biomarkers [13], [18]. In addition, the biological basis for the success or failure of immunotherapies largely depends on the complexity of interactions between tumor cells and immune and immunosuppressive cells in the TME [18], [19], [20]. However, bioinformatics has emerged and proven to be an effective strategy to overcome this challenge via computational extraction of cell type-specific information based on clinical datasets of various cancer types [21], [22], [23]. This strategy is also advantageous because it can accurately capture cell-type-specific profiles and the tissue system level of cell-cell interactions, providing relevant genomic differences for cancer diagnoses, staging, prognoses, and therapeutic responses.

MAX dimerization (MXD) protein 3 (MXD3) is a member of the MXD family of basic-helix-loop-helix-leucine-zipper (bHLHZ) transcription factors that form heterodimers with the MAX cofactor in the MYC/MAX/MXD transcriptional network [24]. Generally, MXD proteins are functional antagonists of MYC, acting as transcriptional repressors to promote cell differentiation [25], [26]; however, MXD3 is an atypical member that has roles in cell cycle progression and cell proliferation rather than differentiation [27], [28], [29], thus act as a tumor promoter. MXD3 is highly expressed in neuroblastoma and medulloblastoma cell lines [30], [31], and it was associated with high-risk features [30]. A previous study has also implicated MXD3 alternative splicing in glioblastoma multiforme (GBM) [32]. In addition, MXD3′s overexpression was shown to promote proliferation in mouse cerebellar granule neuron precursors (GNPs) [29], and to negatively regulate differentiation in mouse spleen-derived B cells [33]. However, Ngo et al. observed that acute activation of MXD3 resulted in a transient increase in cell proliferation, while persistent activation eventually results in decreased cell numbers [31], suggesting that the time course of MXD3 expression dictates cellular outcomes. Therefore, MXD3 appears to be associated with cell proliferation and a variety of human brain cancers. However, insufficient scientific evidence on the pathogenic roles of MXD3 in various cancers and whether MXD3 plays roles in the immune microenvironment and pathogenesis of different tumors through certain common molecular mechanisms and regulates therapy response were the rationalization for the present *in silico* pan-cancer study.

## 2 Materials and methods

### 2.1 Description of the analysis tools

Tumor IMmune Estimation Resource (TIMER2.0) (http://timer.cistrome.org/) [34] is a web server for analyzing gene regulation of the abundance of immune cell infiltration across The Cancer Genome Atlas (TCGA) cancers. GPS-Prot (http://gpsprot.org/index.php) is an online server for visualizing protein–protein interaction (PPI) networks [35]. The OPENTARGET platform (https://www.targetvalidation.org/) integrates genetics, omics, and chemical data to identify the involvement of genes in diseases and aid systematic drug target identification and prioritization [36]. Enrichr (https://maayanlab.cloud/Enrichr/enrich#) is a web server for several enrichment analyses of gene sets [37], [38]. GSCALite (http://bioinfo.life.hust.edu.cn/web/GSCALite/) is an online algorithm for integrating genomic and immunogenomic data of 33 cancer types from TCGA, drug responses from Genomics of Drug Sensitivity in Cancer (GDSC), and normal tissue data from GTEx [39]. The ROC Plotter (http://www.rocplot.org/) is a transcriptome-based tool for predicting biomarkers by linking gene expressions and responses to therapy of breast, ovarian, colorectal, and glioblastoma cancer patients [40].

### 2.2 Data collection

**Fig. 1:**
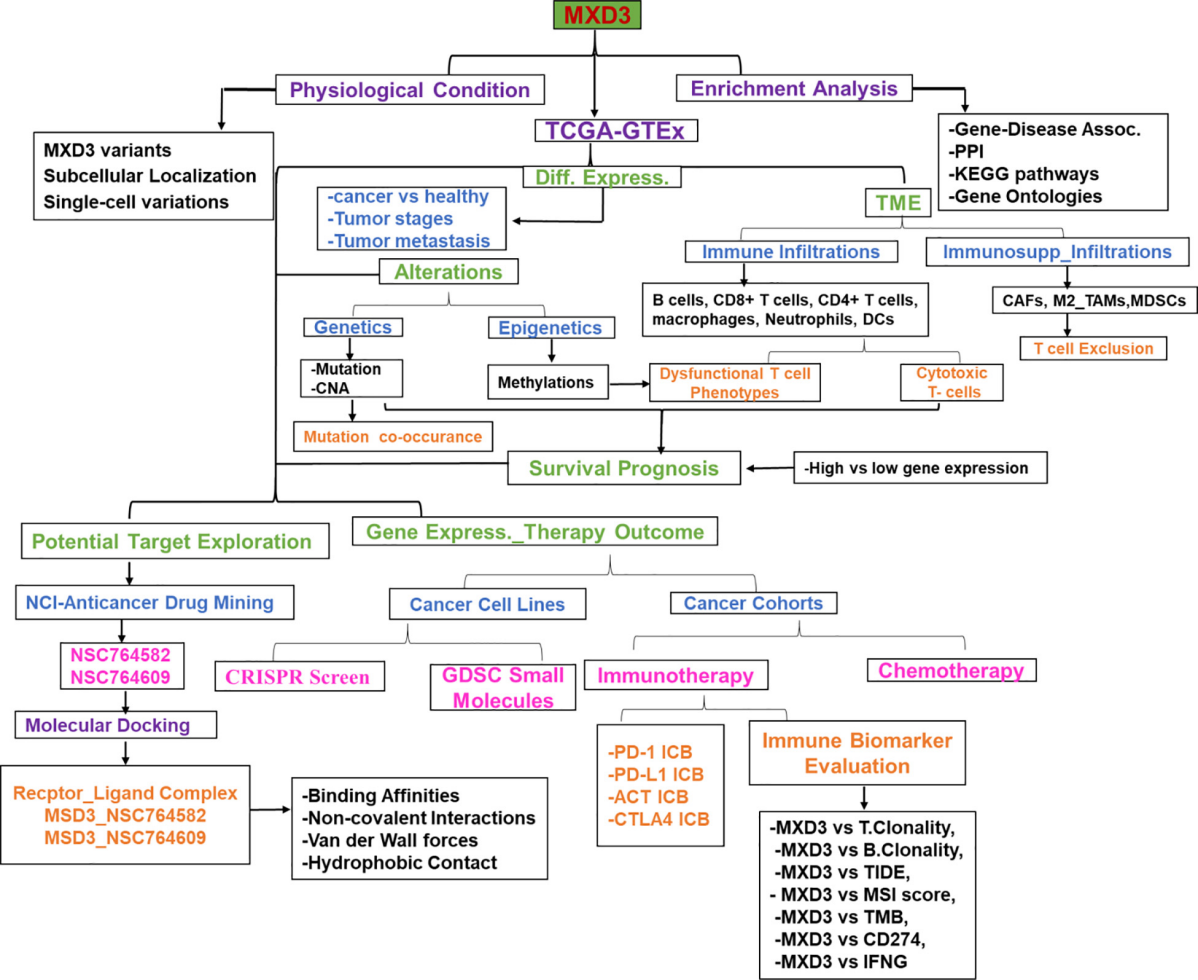
The workflow of the study. GTEx; Genotype-Tissue Expression, KEGG; Kyoto Encyclopedia of Genes and Genomes, PPI, Protein-protein interaction, MXD3; MAX dimerization protein 3, bHLHZ; basic-helix-loop-helix-leucine-zipper, TCGA; The Cancer Genome Atlas, TAMs; M2 subtypes of tumor-associated macrophages, CAFs; cancer-associated fibroblasts, Tregs; regulatory T cells, MDSCs; myeloid-derived suppressor cells, GDSC; Genomics of Drug Sensitivity in Cancer, ICB; Immune checkpoint blockade (ICB) therapy, MSI; Microsatellite instability, TMB; Tumor mutational burden, CD274; Cluster of differentiation 274 IFNG; interferon-γ,

The clinical data of cancer cohorts were obtained from The Cancer Genome Atlas (TCGA) Program of the National Cancer Institute (https://www.cancer.gov/). The list of the 33 TCGA cancer types, their histology, and body location is provided in the Supplementary Material (Table S1). The genomic information from healthy individuals was obtained from The Genotype-Tissue Expression (GTEx) (https://www.gtexportal.org/home/). The GTEx Expression dataset (V8.0) is composed of 17,382 samples from 30 organs (54 tissues), donated by 948 healthy individuals. The workflow of this study is shown in Fig. 1.

### 2.3 Differential MXD3 expression analysis in the normal, tumor, various tumor-stage, and metastatic tissues

To comprehensively analyze differential expression levels of MXD3 between tumor and adjacent normal tissues across TCGA cancer types, we used the TIMER2.0 resource [41], UALCAN interactive web resource [42], and gene expression profiling interactive analysis (GEPIA2) [43] algorithms. To evaluate the differential expression of MXD3 among tumor stages, we used the expression DIY module of GEPIA2 to make a pathological stage (I, II, III, and IV) plot across TCGA cancer types. Furthermore, we used the TNMplot module of the Kaplan-Meier (KM) plotter for a differential gene expression analysis of tumor, normal, and metastatic tissues across various cancers [44]. Differential expression was considered statistically significant at *p* < 0.05, <0.01, and < 0.001.

### 2.4 Analysis of tumor immune and immunosuppressive cell infiltration

We used the TIMER2 server to analyze correlations between MXD3 expression and infiltration of six immune cell types, including B cells, cluster of differentiation 8-positive (CD8^+^) T cells, CD4^+^ T cells, macrophages, neutrophils, and dendritic cells (DCs). We also analyzed correlations of MXD3 expression with tumor infiltration of four immunosuppressive cell types that are known to promote T cell exclusion, viz., myeloid-derived suppressor cells (MDSCs), cancer-associated fibroblasts (CAFs), M2 subtype of tumor-associated macrophages (M2-TAMs), and regulatory T (Treg) cells across 39 TCGA cancer types. A correlation analysis was conducted using the purity-corrected partial Spearman's rho value and statistical significance (*p* < 0.05). We used GraphPad Prism Software (vers. 8.0.0 for Windows) for data visualization. Heatmaps were used to visualize infiltration levels of immune cells across 33 TCGA cancers. In addition, we used the QUERY module the Tumor Immune Dysfunction and Exclusion (TIDE) algorithm to evaluate the effect of genetic and epigenetic alterations of MXD3 on dysfunctional T-cell phenotypes [45].

### 2.5 Gene coexpression and gene alteration co-occurrence analyses

We used the ONCOMINE database (https://www.oncomine.org/resource/login.html) [46] to analyze gene profiles whose expressions were correlated with the expression profile of MXD3 in cancers. The correlation analysis was conducted at a default threshold *p* value of < 10^−04^, a multiple of change of < 2, and gene rank of 10%. In addition, we used the cBioPortal for Cancer Genomics (http://www.cbioportal.org/) to assess gene mutation co-occurrence patterns between MXD3 signatures and other proteins across 10,953 patients from TCGA Pan-Cancer Atlas Studies [47]. A gene alteration co-occurrence analysis was conducted at log ratio of > 5, a *p* value of < 10^−10^, and a q-value of < 10^−10^.

### 2.6 Epigenetic methylation analysis

We used TCGA methylation module [48] of the UALCAN interactive web resource to analyze differential methylation levels of MXD3 between tumor and paired normal tissues across TCGA cancer types. Promoter methylation levels are represented by beta (β) values ranging from 0 (unmethylated) to 1 (fully methylated). Different beta value cutoff points were considered to indicate hypomethylation (β: 0.3–0.25) and hypermethylation (β: 0.7–0.5) [48], [49]. In addition, we analyzed the effects of methylation on dysfunctional T-cell phenotypes and prognoses using the TIDE server.

### 2.7 Analysis of prognostic relevance

To analyze the prognostic relevance of MXD3 differential expression, genetic alterations, and therapeutic outcomes, we used KM curves to analyze overall survival (OS), disease-free survival (DFS), and disease progression-free survival (PFS) of the cohorts. For survival analyses of differentially expressed MXD3 between cancer cohorts, we set the median expression as the expression threshold to split the patient samples into MXD3 high- and low-expression groups, with the hazard ratio (HR), 95% confidence interval (CI), and log-rank test *p* value. All HRs were derived from the Cox proportional hazard regression model and were based on the high versus low comparison.

### 2.8 Functional enrichment and PPI network analysis

We used the GPS-Prot algorithm to conduct the PPI network analysis of MXD3, while the OPENTARGET platform was used to conduct a gene-disease network analysis of MXD3 based on genetic associations. Furthermore, we used MXD3 coexpressed and co-mutated genes to conduct PPI and enrichment analyses. The enrichment analysis of the Kyoto Encyclopedia of Genes (KEGG) pathways and gene ontologies (GOs) were conducted using the Enrich server [37], [38], with the enrichment value set to *p* < 0.05.

### 2.9 Analysis of gene expression correlations with drug sensitivity and therapeutic responses

In order to analyze the effect of MXD3 on therapeutic responses, we used the ROC plotter server to analyze associations of MXD3 transcriptome levels with therapeutic responses in breast, glioblastoma, colorectal, and ovarian cancer patients [40]. In addition, we used the GSCALite server [39] to evaluate the area under the dose–response curve (AUC) values for drugs and gene expression profiles of MXD3 in different cancer cell lines. We then used Spearman correlation coefficients to analyze correlations between MXD3 expression levels and drug sensitivity (50% inhibitory concentration (IC_50_)) to 265 small molecules from the GDSC database.

### 2.10 Gene prioritization and comparative biomarker analysis

We accessed the gene prioritization of MXD3 across two parameters, including the response to immune checkpoint blockade (ICB) therapy and gene-knockout phenotypes in CRISPR screens. The z-score in the Cox-PH regression was used to evaluate the effect of gene expression on patient survival in ICB treatment cohorts. The normalized log(multiple of change [FC]) in CRISPR screens was employed to evaluate the effect of gene knockout-mediated lymphocyte-induced tumor death in cancer models [45]. In addition, the general predictive power of MXD3 in therapeutic response outcomes and OS in different cancer types was compared with seven standardized biomarkers of tumor immune response, including T-cell clonality (T.Clonality), B-cell clonality (B.Clonality), TIDE, estimating the microsatellite instability (MSI) score, tumor mutational burden (TMB), cluster of differentiation 274 (CD274), and interferon-γ (IFNG) using the biomarker evaluation module of the TIDE server [50], [45].

### 2.11 Identification and molecular docking studies of potent MXD3 inhibitors

We used the public COMPARE module of the National Cancer Institute (NCI)-COMPARE program algorithm (https://dtp.cancer.gov/databases_tools/compare.htm) to identify potent anticancer inhibitors of MXD3. The PDB file of the crystal structures of MXD3 (PDB: 6JKK) was obtained from the Protein Data Bank (https://www.rcsb.org/) while the mol2 file of the three-dimensional (3D) structure of the identified compounds (S764609 and S764582) were obtained using the Avogadro molecular builder and visualization tool vers. 1. XX (http://avogadro.cc/) [51], and were converted to PDB files using the PyMOL Molecular Graphics System, vers. 1.2r3pre (Schrödinger; https://pymol.org/edu/?q=educational/). All PDB files were converted to PDBQT files using AutoDock Vina (vers. 0.8, Scripps Research Institute, La Jolla, CA, USA) [52]. The MXD3 was prepared for docking and conducted following standard protocols [15], [53].

## 3 Results

### 3.1 MXD3 variants, localization, single-cell variations, and expression profiles under physiological conditions

**Fig. 2:**
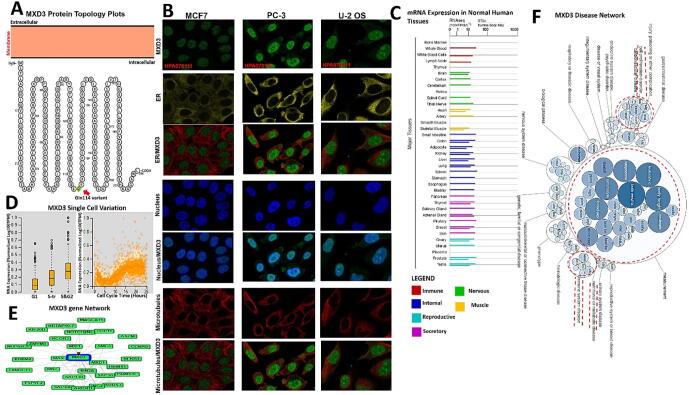
MAX dimerization protein 3 (MXD3) variant, localization, single-cell variations, functional partners, and expression profile under physiological conditions. (A) MXD3 protein topology showing membrane localization with a natural missense variant of Gln114. (B) Immunofluorescence staining of the subcellular distribution of MXD3 within the nucleus, endoplasmic reticulum (ER), and microtubules of PC-3, MCF-7, and U-2 osteosarcoma cells as adopted from the HPA database. (C) Bar plot of MXD3 mRNA expressions in various normal human tissues from the GTEx database. (D) Plots of single-cell RNA-sequencing data from the FUCCI U-2 osteosarcoma cell line, showing the correlation between MXD3 mRNA expression and cell cycle progression. (E) Network of functional gene partners of MXD3 and (F) the MXD3-associated disease network.

The MXD3 protein topology revealed intracellular membrane localization with a natural missense variant of Gln114 [Q(Gln) > H (His)] (Fig. 2A). To characterize the intracellular localization of MXD3, we assessed the distribution of MXD3 within the endoplasmic reticulum (ER) and microtubules of PC-3, MCF-7, and U-2 osteosarcoma (OS) cells using an indirect immunofluorescence assay. We observed that MXD3 colocalized with the nuclear marker in PC-3, MCF-7, and U-2 OS cells, suggesting the subcellular localization of MXD3 in nuclei. In contrast, MXD3 exhibited no overlap with the ER or microtubules in PC-3, MCF-7, and U-2 OS cells (Fig. 2B). Furthermore, we found MXD3 messenger (m)RNA expression in various normal human tissues including the immune, internal, nervous system, secretory, muscle, and reproductive tissues (Fig. 2C). Our analysis of single-cell RNA-sequencing data from Fluorescent Ubiquitination-based Cell Cycle Indicator (FUCCI) U-2 OS cells revealed increased MXD3 RNA expression in relation to cell cycle progression. MXD3 displayed variations in protein expression levels that were temporally correlated with interphase progression through the G_1_, S, and G_2_ phases (Fig. 2D). A gene and disease network interaction analysis revealed that MXD3 has various gene functional partners (Fig. 2E) associated with metabolic diseases, cell proliferation, immune system, and hematological disorders (Fig. 2F).

### 3.2 MXD3 is aberrantly overexpressed and is associated with tumor stages, metastases, and poor cancer prognoses

**Fig. 3:**
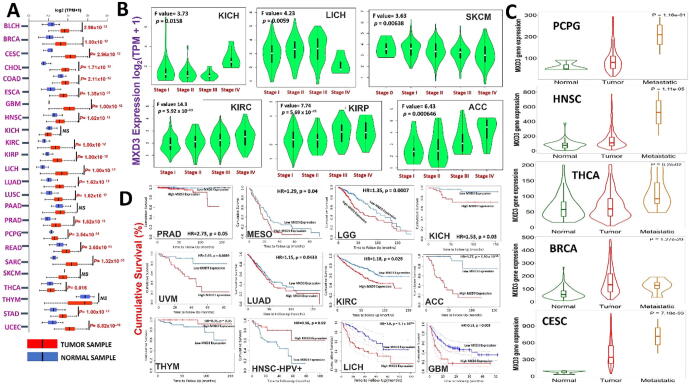
MAX dimerization protein 3 (MXD3) is expressed in multiple cancers in a deregulated manner. (A) Boxplots showing differential MXD3 expression levels (log2 TPM + 1) between tumor and adjacent normal tissues across TCGA database. (B) Violin plots showing differential MXD3 expression levels (log2 TPM + 1) between (B) pathological stages (stages I, II, III, and IV) and (C) metastasis. Only TCGA cancers with statistically significant differences between the pathological stages are presented. (D) Kaplan-Meier curves of cumulative survival differences between TCGA cancer cohorts with high and those with low expression levels of MXD3. Only TCGA cancers with statistically significant differences between the cohorts are presented. TPM: Transcript per million.

We explored the oncogenic role of MXD3 across the TCGA pan-cancer database and found that mRNA levels of MXD3 were significantly (*p* < 0.05) overexpressed in tumors of all TCGA cancer types compared to their corresponding adjacent normal tissues, except for kidney chromophobe (KICH), pancreatic adenocarcinoma (PAAD), and skin cutaneous melanoma (SKCM) (Fig. 3, Supplementary Fig. 1). However, when we compared expression profiles between tumor stages, we found that MXD3 expression increased at higher tumor stages in adrenocortical carcinoma (ACC), KICH, kidney renal clear cell carcinoma (KIRC), kidney renal papillary cell carcinoma, LICH, and SKCM (Fig. 3B). Further, metastatic pheochromocytoma and paraganglioma (PCPG), head and neck squamous cell carcinoma (HNSC), thyroid carcinoma (THCA), breast invasive carcinoma (BRCA), cervical squamous cell carcinoma and endocervical adenocarcinoma (CESC) tumors, exhibited higher expression levels of MXD3 than the corresponding primary tumors (Fig. 3C). However, overexpression of MXD3 was associated with shorter survival of prostate adenocarcinoma (PRAD), mesothelioma (MESO), LGG, KICH, uveal melanoma (UVM), lung adenocarcinoma (LUAD), KIRC, ACC, THYM, HNSC, LICH, and glioblastoma multiforme (GBM) (Fig. 3D). Altogether, these findings strongly suggest that MXD3 is an oncogenic molecule of tumor progression, various tumor stages, and metastasis, and hence could serve as early biomarker for cancer detection, staging, and follow-up.

### 3.3 MXD3 is associated with tumor immune evasion via different mechanisms involving T-cell exclusion in different cancer types and not by tumor infiltration by immune cells

**Fig. 4:**
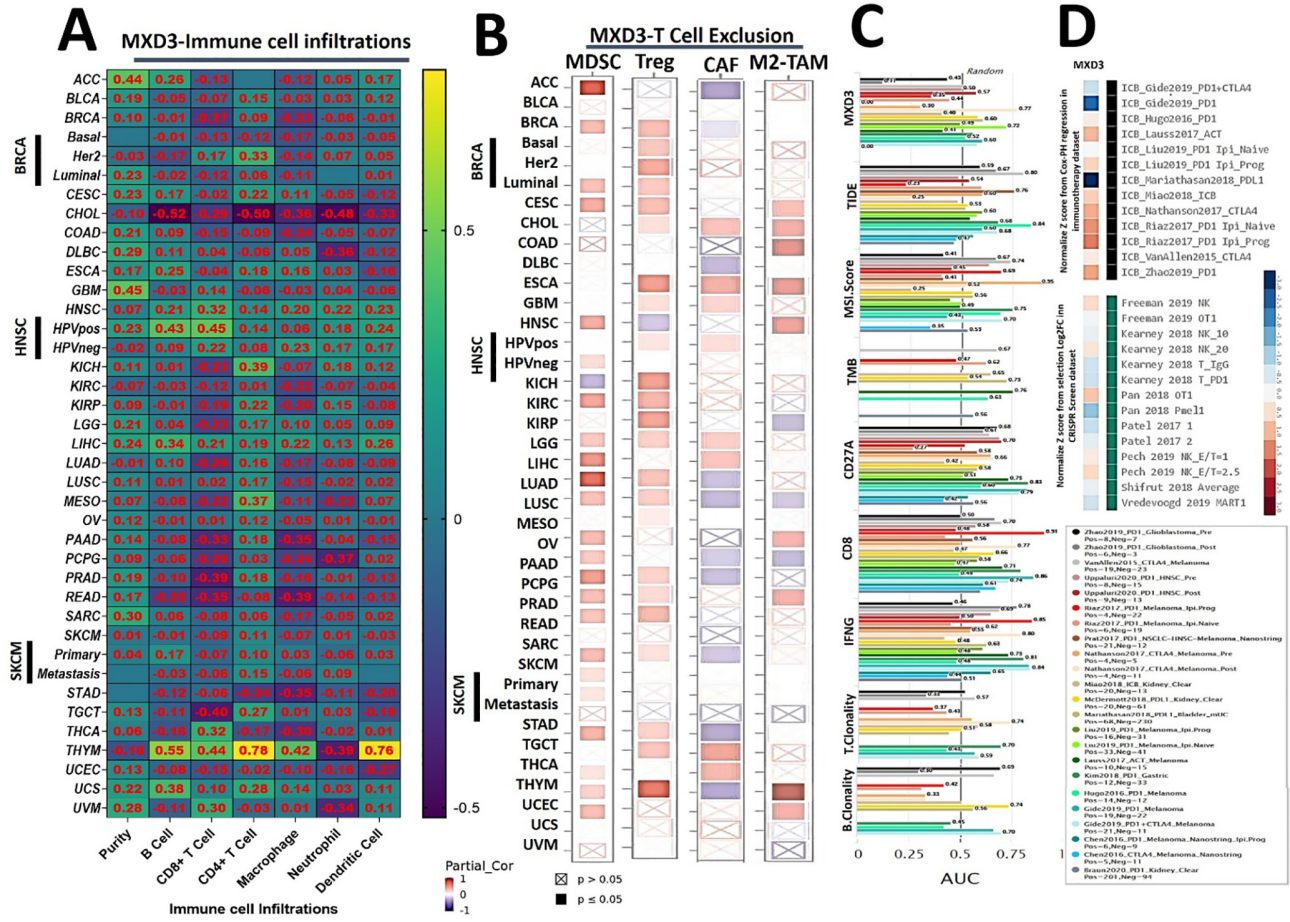
Heatmap showing correlations of MAX dimerization protein 3 (MXD3) expression with infiltration by (A) six immune cell types and (B) four immunosuppressive cell types in various TCGA cancer types. Correlations are depicted with purity-corrected partial Spearman's rho values and statistical significance. (C) Bar plot showing the biomarker relevance of MXD3 compared to standardized cancer immune evasion biomarkers in immune checkpoint blockade (ICB) sub-cohorts. The area under the receiver operating characteristic curve (AUC) was applied to evaluate the predictive performances of the test biomarkers on the ICB response status. (D) Heatmap of MXD3 associations with lymphocyte-mediated tumor killing in CRISPR screens and outcomes in ICB sub-cohorts. CAFs, cancer-associated fibroblasts; MDSCs, myeloid-derived suppressor cells; Tregs, regulatory T cell; M2-TAMs; M2 subtype of tumor-associated macrophages.

Of 39 TCGA cancer types and subtypes evaluated for tumor immune infiltration, only three cancer types (THYM, LICH, and HNSC) showed significant positive correlations of MXD3 expression with infiltration of six immune cell types (B cells, CD8 + T cells, CD4 + T cells, macrophages, neutrophils, and DCs). THYM showed a strong correlation (*r* = 0.42 ∼ 0.77, all *p* < 0.05), while LICH (*r* = 0.12 ∼ 0.33, all *p* < 0.05) and HNSC (*r* = 0.14 ∼ 0.31, all *p* < 0.05) showed week correlations between MXD3 expression levels and tumor infiltration of the six immune cell types, while HNSC-human papillomavirus-positive (HPVpos) showed good correlations of MXD3 expression levels with tumor immune infiltration only of B cells (*r* = 0.43, *p* < 0.05) and CD8 + T cells (*r* = 0.45, *p* < 0.05). All other cancer types showed significant negative associations (*r* < 0, *p* < 0.05) or no significant association (*p* > 0.05) between MXD3 expression levels and tumor infiltration of the six immune cell types (Fig. 4A).

We also assessed correlations of MXD3 expression levels and infiltration of four immunosuppressive cells that are known to promote T-cell exclusion, viz., MDSCs, CAFs, M2-TAMs, and Treg cells. We observed that MXD3 expression was positively correlated with tumor infiltration of MSDCs in ACC, BRCA, BRCA-LumA, BRCA-LumB, GBM, HNSC-HPV, KICH, KIRP, LGG, LIHC, LUAD, MESO, ovarian serous cystadenocarcinoma (OV), PAAD, PCPG, PRAD, SARC, SKCM, STAD, THCA, and UCEC; tumor infiltration of Tregs in BRCA, BRCA-LumA, BRCA-LumB, BRCA-Basal, CESC, DLBC, ESCSA, HNSC, HNSC-HPVpos, KICH, KIRC, KIRP, LICH, LUAD, PAAD, PCPG, PRAD, sarcoma, stomach adenocarcinoma (STAD), testicular germ cell tumor (TGCT), and THYM; tumor infiltration of CAFs in CESC, lymphoid neoplasm diffuse large B-cell lymphoma (DLBC), esophageal carcinoma (ESCA), KIRP, LGG, TGCT, and THCA; and tumor infiltration of M2-TAMs in BRCA-LumA, CESC, cholangiocarcinoma (CHOL), DLBC, GBM, MESO, PCPG, THYM and uterine corpus endometrial carcinoma (UCEC) (Fig. 4B).

We evaluated the biomarker relevance of MXD3 by comparing it with standardized biomarkers based on their predictive power of response outcomes and OS of ICB sub-cohorts. Interestingly, we found that MXD3 alone had an area under the receiver operating characteristic curve (AUC) of > 0.5 in 12 of the 23 ICB sub-cohorts (Fig. 4C). MXD3 exhibited a higher predictive value than TMB, T.Clonality, and B. Clonality, which respectively gave AUC values of > 0.5 in seven, nine, and six ICB sub-cohorts. MXD3 was, however, comparable to the MSI score (AUC > 0.5 in 12 ICB sub-cohorts) but lower than CD27A, TIDE, IFNG, and CD8.

Our results also demonstrated that high expression levels of MXD3 were associated with worse programmed death 1 protein (PD1) outcomes in glioblastoma (ICB_Zhao2019_PD1), in kidney renal clear cell carcinoma (ICB_Miao2018_ICB), PD1 in melanoma (ICB_Riaz2017_PD1), CTLA4 in melanoma (ICB_Nathanson2017_CTLA4), and ACT in melanoma (ICB_Lauss2017_ACT) but achieved good PD-ligand 1 (LI) therapeutic outcomes in bladder (ICB_Mariathasan2018_PDL1) cancer cohorts. At the same time, analysis of gene-knockout phenotypes from genetic screens revealed that MXD3-knockout was a strong influencer of lymphocyte-mediated tumor killing in MC38 colon cancer (Kearney2018_NK_20) and K562 leukemia (Pech2019_NK_E:T = 2.5) models (Fig. 4D).

### 3.4 Epigenetic modification of MXD3 is associated dysfunctional T-cell phenotypes and poor prognoses of cancer cohorts

**Fig. 5:**
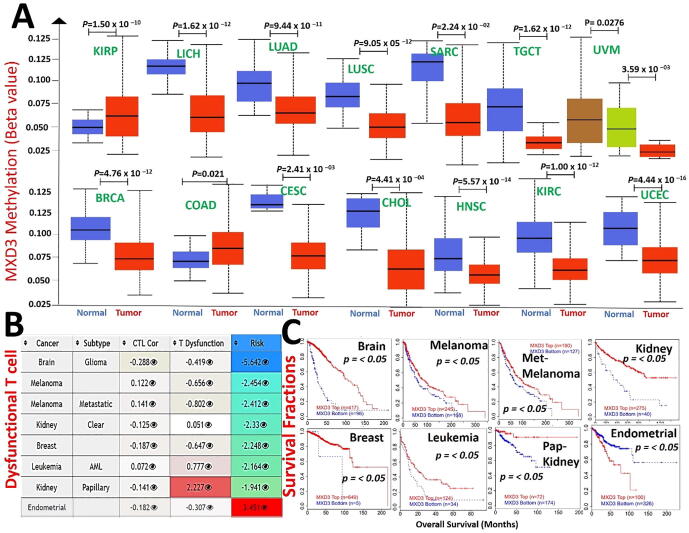
Epigenetic modification of MAX dimerization protein 3 (MXD3) mediates dysfunctional T-cell phenotypes and poor prognoses of cancer cohorts. (A) Boxplots showing differential MXD3 methylation levels (beta values) between tumor and adjacent normal tissues across TCGA database. (B) Heatmap showing the roles of MXD3 methylation in cytotoxic T-cell levels (CTLs), dysfunctional T-cell phenotypes, and risk factors of TCGA cancer cohorts. (C) Kaplan-Meier curves of overall survival differences between TCGA cancer cohorts with high methylation levels and those with low methylation levels of MXD3. Only TCGA cancers with statistically significant differences between cohorts are presented.

Analysis of the promoter methylation status revealed that MXD3 is hypermethylated in KIRP. At the same time, it is hypomethylated in various cancer types, including LICH, LUAD, lung squamous cell carcinoma (LUSC), SARC, TGCT, UVM, BRCA, colon adenocarcinoma (COAD), cervical squamous cell carcinoma, and endocervical adenocarcinoma (CESC), CHOL, HNSC, KIRC, and UCEC (Fig. 5A). These findings indicate that MXD3 methylation is inversely associated with mRNA expression levels in various cancers (*r* = 0.1 ∼ 0.48, all *p* < 0.05, Supplementary Table S2); hence, we evaluated the consequences of the MXD3 methylation status in various cancers. Interestingly, we found that hypomethylation of MXD3 was positively associated with dysfunctional T cell phenotypes (Fig. 5B) and shorter survival durations of the brain, melanoma, metastatic melanoma, leukemia, breast cancer, and KIRC cohorts. In contrast, hypomethylation of MXD3 was associated with a good prognosis in endometrial cancer. In accordance with the differential methylation status of MXD3 in KIRP, hypermethylation in KIRP was positively associated with dysfunctional T-cell phenotypes but was associated with longer survival of kidney papillary carcinoma cohorts (Fig. 5C). Collectively, these findings indicate that epigenetic methylation of MXD3 in cancer patients is associated with dysfunctional T-cell phenotypes via different mechanisms that ultimately result in poor prognoses of melanoma, leukemia, breast, glioma, and kidney cancer cohorts while prolonging the survival of endometrial cancer cohorts.

### 3.5 Genetic alterations and oncogenic features of MXD3 co-occur with deregulation of its functional partner proteins and are associated with poor prognoses of cancer cohorts

**Fig. 6:**
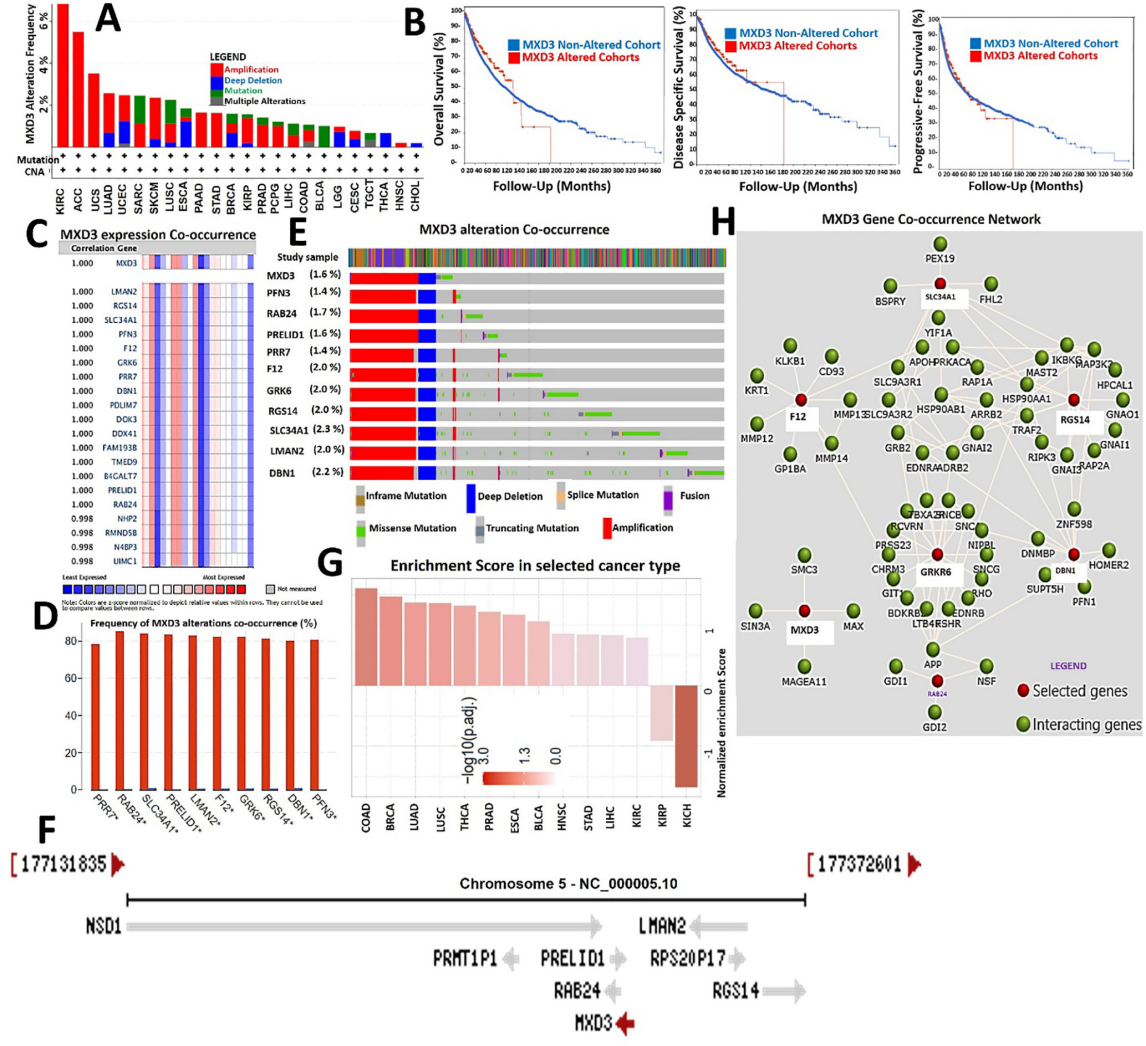
Genetic alterations and oncogenic features of MAX dimerization protein 3 (MXD3) co-occurred with deregulation of its functional partner proteins and were associated with poor prognoses of cancer cohorts. (A) Bar plot of MDX3 alteration frequencies across various cancer types. (B) Kaplan-Meier curves of differences in overall survival, disease-specific, and progression-free survival between cancer cohorts with altered MXD3 and those without altered MXD3. (C) Heatmap showing MXD3 over**e**xpression co-occurrence. The strength of correlations between the genes is reflected by the partial Spearman's rho value and estimated statistical significance, where a value of *r* = 1 means a perfect positive correlation. (D) Bar plot showing the frequencies of *DBN1*, *RAB24*, *SLC34A1*, *PRELID1*, *LMAN2*, *F12*, *GRK6*, *RGS14*, *PRR7*, and *PFN3* alteration co-occurrence with MXD3 alterations. (E) Waterfall plot showing the co-occurrence pattern of MXD3 alterations with genetic alterations of *DBN1*, *RAB24*, *SLC34A1*, *PRELID1*, *LMAN2*, *F12*, *GRK6*, *RGS14*, *PRR7*, and *PFN3*. (F) MXD3 gene co-occurrence (G) enrichment in various cancer types, and (H) protein–protein interaction network of MXD3 gene co-occurrence.

We queried the frequencies and types of genetic alterations of MXD3 across the various cancer types and found that gene amplification was the most frequent genetic alteration of MXD3 followed by MXD3 mutations and deep deletions, while multiple alterations of MXD3 were less frequent (Fig. 6A). However, these genetic alterations were associated with the cohorts' OS, DFS, and PFS (Fig. 6B). We conducted a gene correlation analysis and found that MXD3 expression in cancer cohorts was near-perfectly correlated (*r* ≥ 0.99) with expression levels of several other genes, including *DBN1*, *RAB24*, *SLC34A1*, *PRELID1*, *LMAN2*, *F12*, *GRK6*, *RGS14*, *PRR7*, and *PFN3* (Fig. 6C). Similarly, genetic alterations of MXD3 co-occurred with the frequency and pattern of genetic alterations of these same genes (Fig. 6D, E), suggesting that these genes are functional partners associated with the oncogenic role of MXD3 in various cancer types. Interestingly, these genes share similar chromosome locations with MXD3 (Fig. 6F). In order to unravel the biological processes mediated by this collection of genes, we constructed PPI and gene enrichment network analyses (Fig. 6G, H). We found that the major biological processes regulated by this collection of genes were “inorganic anion homeostasis” and “regulation of phospholipid transport,” while KEGG pathways included “the Rap1 signaling pathway”, “chemokine signaling,” and “complement and coagulation cascades” (Table 1). The enrichment analysis revealed that gene signatures were primarily enriched in COAD, BRCA, LUAD, LUSC, THCA, PRAD, ESCA, BLCA, HNSC, STAD, LICH, KIRC, and KIRP. Collectively, our findings suggest that the oncogenic roles of MXD3 are concomitantly associated with deregulation of *DBN1*, *RAB24*, *SLC34A1*, *PRELID1*, *LMAN2*, *F12*, *GRK6*, *RGS14*, *PRR7,* and *PFN3* in various cancer types.

**Table 1:** Enriched pathways and gene ontologies associated with MAX dimerization protein 3 (MXD3) and functional protein partners.

Index	KEGG pathways	*p* value	Adjusted *p* value	Odds ratio	Combined score
1	Rap1 signaling pathway	0.005462	0.05462	21.55	112.29
2	Shigellosis	0.03518	0.09465	31.13	104.21
3	Complement and coagulation cascades	0.04261	0.09465	25.53	80.55
4	Salmonella infection	0.04631	0.09465	23.42	71.95
5	Morphine addiction	0.04894	0.09465	22.11	66.71
6	Parathyroid hormone synthesis, secretion, and action	0.05679	0.09465	18.94	54.32
7	Protein processing in endoplasmic reticulum	0.08712	0.1240	12.09	29.50
8	Chemokine signaling pathway	0.09970	0.1240	10.48	24.15
9	Regulation of actin cytoskeleton	0.1116	0.1240	9.28	20.36
10	Endocytosis	0.1263	0.1263	8.13	16.81

Index	Biological processes	*p* value	Adjusted *p* value	Odds ratio	Combined score
1	Response to lead ions (GO:0010288)	0.003296	0.03288	399.68	2284.21
2	Negative regulation of membrane potential (GO:0045837)	0.003296	0.03288	399.68	2284.21
3	Positive regulation of phospholipid transport (GO:2001140)	0.003296	0.03288	399.68	2284.21
4	Response to misfolded proteins (GO:0051788)	0.003844	0.03288	333.05	1852.16
5	Cellular phosphate ion homeostasis (GO:0030643)	0.003844	0.03288	333.05	1852.16
6	Cellular divalent inorganic anion homeostasis (GO:0072501)	0.003844	0.03288	333.05	1852.16
7	Positive regulation of lymphocyte apoptotic process (GO:0070230)	0.003844	0.03288	333.05	1852.16
8	Cellular trivalent inorganic anion homeostasis (GO:0072502)	0.003844	0.03288	333.05	1852.16
9	Positive regulation of lipid transport (GO:0032370)	0.003844	0.03288	333.05	1852.16
10	Cellular monovalent inorganic anion homeostasis (GO:0030320)	0.004392	0.03288	285.46	1549.44

### 3.6 MXD3 expression is associated with therapeutic responses in multiple cancer types

**Fig. 7:**
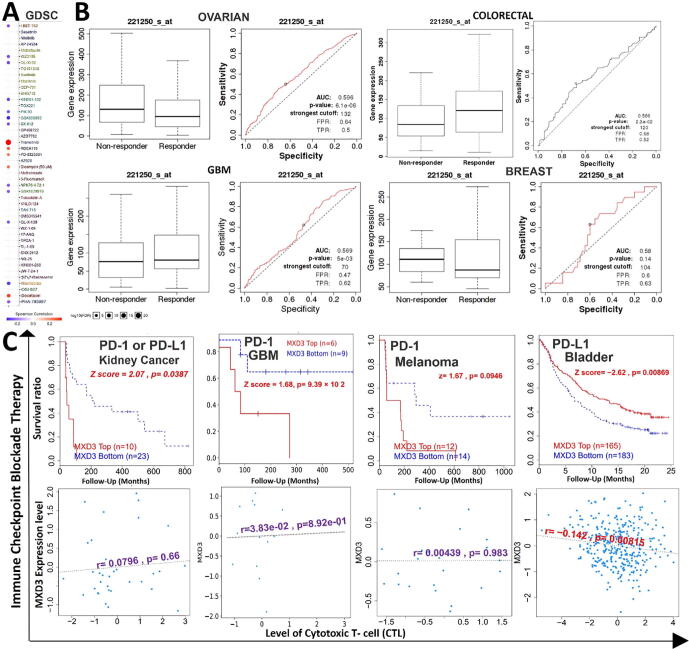
MAX dimerization protein 3 (MXD3) expression is associated with therapeutic responses in multiple cancer types. (A) Bubble plot of the correlation between the sensitivity of drugs in the Genomic of Drug Sensitivity in Cancer (GDSC) database and mRNA expression of MXD3. Colors from blue to red represent the correlations between mRNA expression and 50% inhibitory concentration (IC_50_) values. A positive correlation means that the gene's high expression was resistant to the drug and vice versa. The bubble size was positively correlated with false detection rate (FDR) significance. (B) The receiver operating characteristic (ROC) curve plot of the association between MXD3 expression and responses to chemotherapy in breast, brain, colorectal, and ovarian cancer cohorts. (C) Kaplan-Meier curves (upper panel) of survival ratios as a measure of the immunotherapeutic response (immune checkpoint blockade) between cancer cohorts with high and those with low expression levels of MXD3. The lower panel shows the correlation between the MXD3 expression and cytotoxic T-cell level (CTL) in these cohorts. Only TCGA cancers with statistically significant differences between the cohorts are presented. (For interpretation of the references to colour in this figure legend, the reader is referred to the web version of this article.)

We analyzed associations between MXD3 expression and activities of different clinical chemotherapies on various cancer cell lines. We found that higher expression levels of MXD3 were associated with decreased sensitivity of cancer cell lines to trametinib (a MEK inhibitor), docetaxel, RDEA119 (a MEK inhibitor), PD-0325901 (a MEK inhibitor), and bleomycin but led to increased activities of I-BET-762, WZ3105, QL-XI-92, KIN001-102, GSK690693, GSK1070916, NPK76-II-72–1, QL-X-138, and navitoclax in various cancer cell lines (Fig. 7A). Furthermore, we evaluated the effect of MXD3 expression on chemotherapeutic responses in clinical cancer cohorts. We found that glioblastoma, ovarian, and breast cancer patients with higher MXD3 expressions were resistant to chemotherapies, while colorectal cancer patients with higher MXD3 expression benefited more from chemotherapies than cohorts with lower expression (Fig. 7B). In addition, we found that lower expression levels of MXD3 were associated with clinical benefits of ICB therapy (PD-1 or PD-L1) in melanomas, glioblastomas, and kidney cancer and hence exhibited prolonged survival periods compared to cohorts with high MXD3 expression levels (Fig. 7C, upper panel). In contrast, higher MXD3 expression was associated with clinical benefits in bladder cancer patients to PD-L1 ICB and hence exhibited higher survival durations than bladder cancer cohorts that had lower MXD3 expression levels. However, increased expression levels of MXD3 in bladder cancer cohorts were negatively associated with the level of CTL, suggesting an interplay with T cell exclusion (Fig. 7C, lower panel).

### 3.7 *In silico* identification of potent anticancer inhibitors of the MXD3 signaling axis

**Fig. 8:**
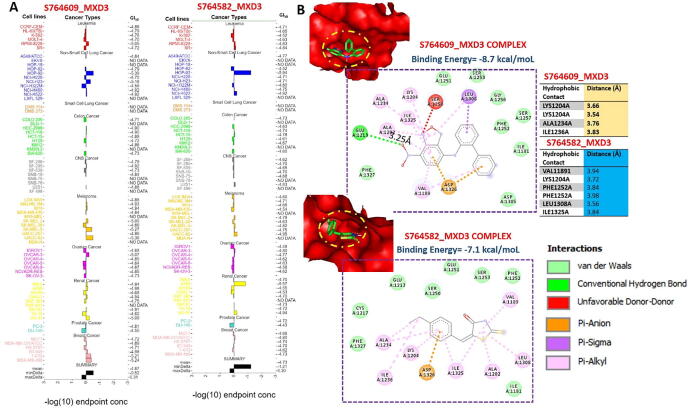
*In silico* identification of potent inhibitors of the MAX dimerization protein 3 (MXD3) signaling axis. (A) 50% growth inhibition (GI_50_) concentrations of potent inhibitors of the MXD3 signaling axis against panels of NCI cancer cell lines. (B) Dose-dependent anticancer activities of potent inhibitors of the MXD3 signaling axis against panels of NCI cancer cell lines. (C) Molecular docking profile of S764609 and S764582 with MXD3.

We used the COMPARE algorithm of the NCI Development Therapeutics Program (DTP) to identify potent anticancer drugs whose specific mechanism of action involved the inhibition of MXD3 signaling. Surprisingly, out of the over 88,000 pure compounds reported for anticancer activities against panels of NCI cancer cell lines, we identified only two synthetic compounds, (5E)-5-[(4-ethylphenyl) methylidene]-2-sulfanylidene-1,3-thiazolidin-4-one (S764582) and 7-nitro-N-(2-phenylphenyl)-2,1,3-benzoxadiazol-4-amine (S764609), that specifically targeted the MXD3 signaling pathway. These compounds were respectively reported to inhibit the growth of 58 and 59 cancer cell lines, representative of leukemia, melanoma, prostate, renal, ovarian, CNS, NSCLC, and colon cancers with GI_50_ concentrations (-log10) of −4.87 ± 0.19 and 4.73 ± 0.22 μM for S764609 and S764582, respectively (Fig. 8A). In order to gain more mechanistic insights into interactions between these compounds and MXD3, we conducted a molecular docking study and found that both S764609 and S764582 fit into the binding cavity of MXD3 with respective binding affinities of −8.7 and −7.1 kcal/mol. The compounds bonded with MXD3 by hydrogen bonds, pi-interactions, alkylation, and several van der Waals forces. In addition, there are several hydrophobic contacts between the complex (Fig. 8B).

## 4 Discussion

Our results revealed that MXD3 is closely related to the occurrence and development of various systemic diseases and cancers. Studies also reported functional links between MXD3 and clinical diseases, especially tumors [31], [30], [54]. Whether MXD3 plays a role in the immune microenvironment and pathogenesis of different tumors through specific or common molecular mechanisms remains to be resolved. This study demonstrated the relationships of expression levels and genetic or epigenetic alterations of MXD3 with tumor staging, metastasis, TME, immune evasion, and drug sensitivity across 39 TCGA cancer types and subtypes.

Cancers of different types and subtypes exhibited distinct genetic heterogeneity and clinical features [55], [56]. Protein profiling can provide valuable insights into identifying prognostic biomarkers for early diagnosis and facilitate treatment designs for particular cancer types and subtypes [57], [58]. In this study, we explored the oncogenic role of MXD3 across TCGA pan-cancers. We found that mRNA levels of MXD3 were expressed in a deregulated manner in almost all TCGA cancer types and were correlated with tumor staging or metastasis of ACC, KICH, KIRC, KIRP, LICH, SKCMC of PCPG, HNSC, THCA, BRCA, and CESC; these findings suggested that MXD3 is an oncogenic molecule of tumor progression, invasion, and metastasis. Thus, MXD3 could serve as an early biomarker for cancer detection and follow-up. Nevertheless, data from the survival prognosis analysis of the *MXD3* gene suggested distinct conclusions for different tumors, as we observed that *MXD3* predicted worse survival of PRAD, MESO, LGG, KICH, UVM, LUAD, KIRC, ACC, THYM, HNSC, LICH, and GBM cohorts. Our findings are consistent with previous clinical and preclinical studies, which demonstrated that MXD3 was highly expressed in neuroblastoma and medulloblastoma cells [30], [31], and it was associated with high-risk features [30]. In accordance with our findings, Ngo et al. [32] reported that MXD3 alternative splicing is associated with the differential mRNA stability between splice variants with consequent pathogenic implications in GBM. In addition, MXD3 overexpression was shown to promote proliferation in mouse cerebellar granule neuron precursors (GNPs) [29] and negatively regulate differentiation in spleen-derived mouse B cells [33]. However, Ngo et al. [31] observed that acute activation of MXD3 resulted in a transient increase in cell proliferation, while persistent activation eventually resulted in decreased cell number [31], suggesting that the time course of MXD3 expression dictates cellular outcomes.

In the present study, we failed to detect a correlation between MXD3 expression and survival prognosis of BLCA, BRCA, CESC, CHOL, COAD, DLBC, ESCA, KIRP, LAML, LUSC PRAD, THYM, UCEC, or UCS patients; hence, the current TCGA clinical data-based evidence cannot support the role of MXD3 overexpression in the clinical prognosis of those cancers. We, therefore, postulated the possibility that high MXD3 expression levels in those cancer types are a byproduct of dysregulated signaling without prognostic significance for tumor cells. However, in contrast, as discussed below, our further analysis suggested the involvement of MXD3 genetic and epigenetic alterations in regulating the tumor immune microenvironment and worse clinical prognoses of cohorts of those cancers.

The TME and tumor immune evasion are correlated with cancer prognoses and therapeutic [59]. However, two distinct mechanisms of immune evasion were proposed. First, tumor infiltration of immune cells leads to T-cell anergy or dysfunctional T-cell phenotypes [60], [61], which promote tumor escape of the host immune system, tumor progression, invasion, metastasis, and therapeutic resistance [61]. Studies indicated that aberrant infiltration of immune cells into normal tissues might also enhance tumor development and progression [62]. Studies indicated that some oncogenic proteins regulate tumor infiltration of immune cells. However, our exploration of MXD3′s association with tumor immune infiltration across 39 TCGA databases indicated that MXD3 is associated with tumor infiltration of immune cells only in THYM, LICH, and HNSC, suggesting that MXD3 could enhance tumor immune evasion and progression of THYM, LICH, and HNSC via dysfunctional T cell phenotypes.

The second mechanism of tumor immune evasion is the T-cell exclusion mechanism [63], whereby the tumor prevents infiltration of immune cells. T-Cell exclusion is dependent on the infiltration of immunosuppressive cells such as CAFs, Tregs, M2-TAMs, and MDSCs and is therefore known as biomarkers of T-cell exclusion in cancer [64]. Interestingly, we found that MXD3 expression levels correlated well with expressions of immunosuppressive cells in almost all cancer types. Therefore, we speculated that T-cell exclusion is the major mechanism through which MXD3 regulates tumor escape of immune cells, tumor promotion, and metastasis. However, the involvement of tumor infiltration of immune cells in THYM, LICH, and HNSC suggests that MXD3 exhibits a tissue-dependent mechanism of regulating immune evasion. Moreover, we found that MXD3 is hypomethylated in various cancer types, except KIRP. The differential methylation statuses of MXD3 in TCGA cancers were negatively correlated with differential mRNA overexpression levels in those cancers, suggesting that epigenetic methylation of MXD3 could affect the transcriptome of TCGA tumor cells. Intriguingly, our results demonstrated that hypomethylation of MXD3 mediated dysfunctional T-cell phenotypes and shorter life durations of brain cancer, melanoma, metastatic melanoma, leukemia, breast cancer, and kidney renal clear cell carcinoma (KIRC) cohorts. However, our findings indicated that the methylation status of MXD3 mediated dysfunctional T-cell phenotypes and worse prognoses of KIRP and endometrial cancer via a mechanism that differs from other cancer types. This further supports our hypothesis that MXD3 exhibits a tissue-dependent mechanism of regulating immune evasion and patient prognoses.

The accumulation of genetic alterations drives the progression of normal cells through hyperplastic and dysplastic stages to invasive cancer and, ultimately, metastatic disease [65]. Gene alteration analyses of known oncogenes should therefore provide further insights into the roles of these genes in cancer progression [66]. Consequently, we queried the types and genetic alteration frequencies of MXD3 across various cancer types and found that gene amplification and mutations were the most frequent genetic alterations of MXD3, while deep deletions occurred less frequently. As genetic alterations in tumors are common, changes found in premalignant stages are more likely to represent pivotal events initiating and promoting cancer development [67]. These events may be masked by complex patterns of genetic alterations often associated with genetic instability in later disease stages. Therefore, to fully understand how malignant tissues develop, all stages of progression must be considered [67].

Interestingly, genetic alterations of MXD3 were associated with worse prognoses in the context of OS, DFS, and PFS. These findings, together with the overall expression profiles of MXD3 in primary tumors and pathological and metastatic stages, suggest its involvement at all stages of tumor progression, and hence it can serve as an attractive target for cancer therapies. However, cancer development and progression cannot be attributed to a single gene, as co-occurrences of gene alterations are frequently observed and conjoin with the primary genetic driver as co-drivers to promote tumor progression and limit therapeutic responses [68], [69]. Therefore, we analyzed gene expression and alteration co-occurrence to identify functional partners of MXD3 in cancers. Fascinatingly, we found near-perfect correlations (*r* ≥ 0.99) between the MXD3 expression level and expression levels of *DBN1*, *RAB24*, *SLC34A1*, *PRELID1*, *LMAN2*, *F12*, *GRK6*, *RGS14*, *PRR7*, and *PFN3* in cancers. Our search for co-occurring genetic alterations also revealed that these same genes exhibited very high (>80%) enrichment with MXD3 in the frequency and pattern of genetic alterations, suggesting that these genes are functional partners associated with the oncogenic role of MXD3 in various cancer types.

Furthermore, we integrated MXD3 with these co-functional partners across all tumors for enrichment analyses and identified enrichment of “inorganic ion homeostasis,” “regulation of phospholipid transport,” “chemokine signaling,” and “complement and coagulation cascades” in the MXD3 network-mediated pathogenesis of various cancers. Alterations in iron metabolism are among metabolic and immunological hallmarks of cancer [70] because cancer largely depends on iron for proliferation. Therefore, our results suggested that MXD3 and its co-functional proteins exhibited oncogenic roles via activating membrane phospholipids and ion metabolism-related signaling pathways. Furthermore, in line with our observation that MXD3 is associated with tumor immune evasion and the involvement of MXD3 co-functional proteins in chemokine signaling and complement and coagulation cascades, studies also indicated that altered iron homeostasis can modulate immune responses in favor of cancer progression [71], [72]. Further experimental studies are, however, required to unravel how MXD3 mediates the involvement of ion homeostasis in tumor development and progression.

Antibodies against PD-1 or PDL-1 effectively treat a variety of cancers and improve prognoses [73]. Our analysis revealed that the melanoma, glioblastoma, and kidney cancer cohorts with low MXD3 expression exhibited higher clinical benefits of ICB therapy (PD-1 or PD-L1). In contrast, higher MXD3 expression was associated with the clinical benefits of PD-L1 in bladder cancer. Furthermore, increased expression levels of MXD3 in these cohorts were inversely correlated with CTLs, suggesting an interplay of T-cell exclusion according to our previous observations. In addition, upon further exploration of the critical role of MXD3 in predicting therapeutic responses, we analyzed associations of MXD3 expression with activities of various clinical chemotherapies on cancer cell lines. Surprisingly, we found that higher expression levels of MXD3 were associated with decreased sensitivity of cancer cell lines to several MEK inhibitors but led to increased activities of other kinase inhibitors, including Akt inhibitors. The reason behind this selectivity of MXD3 in mediating drug activities merits further investigation.

Our analysis also revealed that glioblastoma, ovarian, and breast cancer patients with higher MXD3 expressions were resistant to chemotherapies. In accordance with our observations, previous studies reported that MXD3-knockdown resulted in apoptosis of neuroblastoma cells and enhanced the therapeutic efficacy of chemotherapies against a neuroblastoma cell line [54] while knocking down/target deletion of MXD3 induced apoptosis of Reh human precursor B acute lymphoblastic leukemia cells [54] and sensitized neuronal and lymphoid cells to radiation-induced apoptosis [74]. Altogether, our study strongly suggests that MXD3 is an immune-oncogenic molecule and could serve as a biomarker for cancer detection, prognosis, therapy design, and follow-up. To our delight, through the mining of the NCI-DTP database, we identified two synthetic compounds (5E)-5-[(4-ethylphenyl) methylidene]-2-sulfanylidene-1,3-thiazolidin-4-one (S764582) and 7-nitro-N-(2-phenylphenyl)-2,1,3-benzoxadiazol-4-amine (S764609) that specifically target the MXD3 signaling pathway. These compounds fit well into the binding cavity and demonstrated robust interactions with MXD3, and are currently under vigorous preclinical investigation in our laboratory.

## 5 Conclusions

In conclusion, our results suggest that MXD3 plays pivotal pathogenic roles in the immuno-oncology context of the TME, prognoses, and therapeutic responses through different mechanisms involving T-cell exclusion, tumor infiltration of immune cells in THYM, LICH, and HNSC. Genetic alterations and epigenetic modifications of MXD3 were associated with poorer prognoses. Oncogenic features of MXD3 were concomitantly associated with deregulation of *DBN1*, *RAB24*, *SLC34A1*, *PRELID1*, *LMAN2*, *F12*, *GRK6*, *RGS14*, *PRR7*, and *PFN3* and were connected to phospholipid transport and ion homeostasis. Collectively, our study suggests that MXD3 could serve as a biomarker for cancer detection, prognosis, therapy design, and follow-up.

## 6 Author contributionss

SY Wu, and KC Lin helped with data collection; BL wrote the manuscript; ATH Wu and CZ Wu designed and oversaw the study.

## 7 Fundingss

ATH Wu was supported by research grants by Taipei Medical University and the Ministry of Education, Taiwan (DP2-110–21121-03-C-09 and DP2-110–21121-01-H-03–03). ATH Wu and SY Wu were funded by the Lotung Poh-Ai Hospital research grant (grant no. 11002). KC Lin and CZ Wu were funded by Wan Fang Hospital, Taipei Medical University research grant (110-phd-04).

## 8 Institutional Review Board Statement

Not applicable.

## 9 Informed Consent Statement

Not applicable.

## 10 Data Availability Statement

The raw data supporting the conclusions of this article are available upon reasonable request.

Not applicable.

## CRediT authorship contribution statement

**Szu-Yuan Wu:** Conceptualization, Data curation, Resources, Software, Writing - review & editing. **Kuan-Chou Lin:** Conceptualization, Data curation, Software, Writing – original draft. **Bashir Lawal:** Conceptualization, Data curation, Software, Writing – original draft, Writing - review & editing. **Alexander T.H Wu:** Conceptualization, Funding acquisition, Resources, Supervision, Visualization, Writing – original draft, Writing - review & editing. **Ching-Zong Wu:** Conceptualization, Funding acquisition, Software, Supervision, Visualization, Writing – original draft.

## Declaration of Competing Interest

The authors declare that they have no known competing financial interests or personal relationships that could have appeared to influence the work reported in this paper.
