# Supplementary material for: MXD3 as an onco-immunological biomarker encompassing the tumor microenvironment, disease staging, prognoses, and therapeutic responses in multiple cancer types
Source: Comput Struct Biotechnol J. 2021 Sep 2;19:4970–83. doi: 10.1016/j.csbj.2021.08.047 (PMC8441106; doi:10.1016/j.csbj.2021.08.047)
Supplement: Supplementary data 1 [file mmc1.docx]

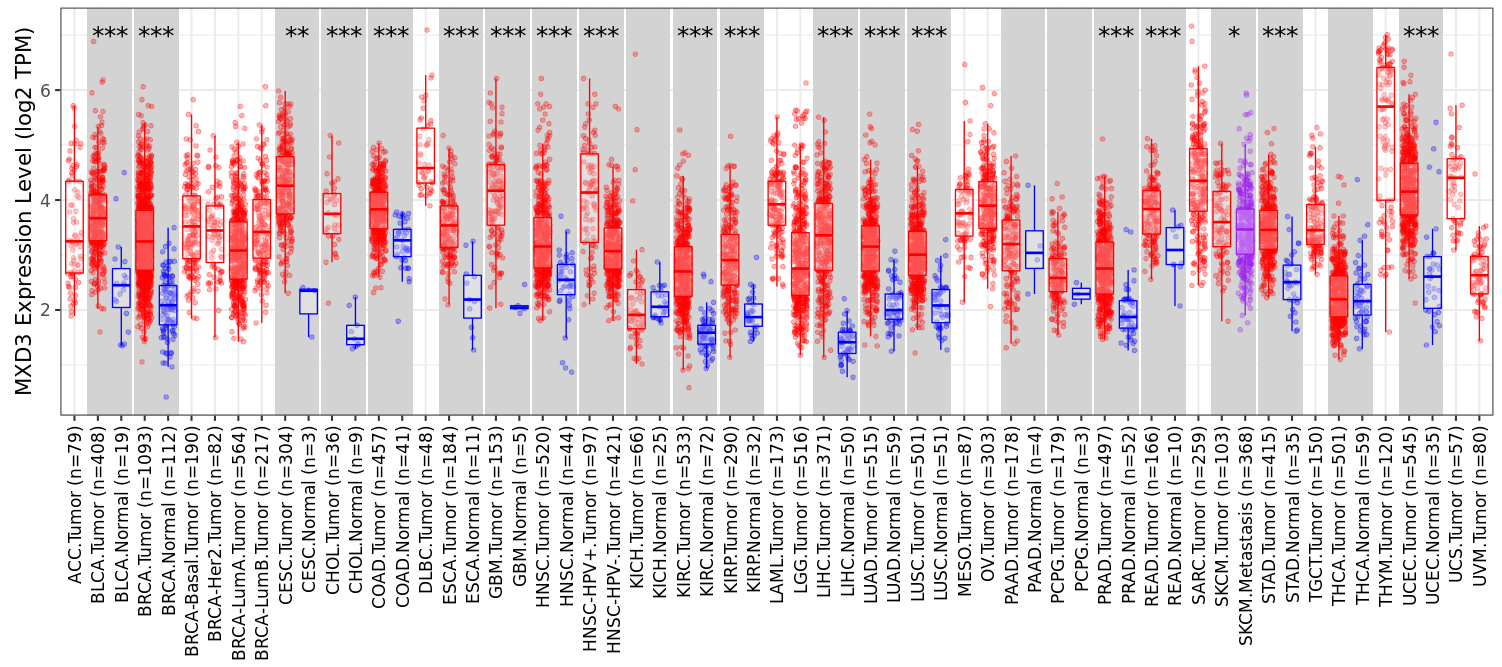


Figure S1: differential MXD3 expression across various cancer types.

**Table S1.** List of pan-cancer analyzed in this study.

| **TCGA code** | **Cancer type** | **Histology** | **Body Location** |
| --- | --- | --- | --- |
| ACC | Adrenocortical carcinoma | Carcinomas | Endocrine |
| BLCA | Bladder urothelial carcinoma | Carcinomas | Genitourinary |
| BRCA | Breast invasive carcinoma | Carcinomas | Breast |
| CESC | Cervical squamous cell carcinoma and endocervical adenocarcinoma | Carcinomas | Gynecology |
| CHOL | Cholangiocarcinoma (bile duct) | Carcinomas | Digestive |
| COAD | Colon adenocarcinoma | Carcinomas | Digestive |
| DLBC | Lymphoid neoplasm diffuse large B−cell lymphoma | lymphoma | Lymphoma |
| ESCA | Esophageal carcinoma | Carcinomas | Digestive |
| GBM | Glioblastoma multiforme | sarcomas | Neurologic |
| HNSC | Head and neck squamous cell carcinoma | Carcinomas | Head and neck |
| KICH | Kidney chromophobe | Carcinomas | Genitourinary |
| KIRC | Kidney renal clear cell carcinoma | Carcinomas | Genitourinary |
| KIRP | Kidney renal papillary cell carcinoma | Carcinomas | Genitourinary |
| LAML | Acute myeloid leukemia | Leukemia | Hematologic |
| LGG | Brain lower grade glioma | Sarcoma | Neurologic |
| LIHC | Liver hepatocellular carcinoma | Carcinomas | Digestive |
| LUAD | Lung adenocarcinoma | Carcinomas | Respiratory |
| LUSC | Lung squamous cell carcinoma | Carcinomas | Respiratory |
| OV | Ovarian serous cystadenocarcinoma | Carcinomas | Gynecology |
| PAAD | Pancreatic adenocarcinoma | Carcinomas | Digestive |
| PCPG | Pheochromocytoma and paraganglioma (adrenal gland) |  | Endocrine |
| PRAD | Prostate adenocarcinoma | Carcinomas | Genitourinary |
| READ | Rectum adenocarcinoma | Carcinomas | Digestive |
| SARC | Sarcoma | Sarcoma | Gynecology |
| SKCM | Skin cutaneous melanoma |  | Skin |
| STAD | Stomach adenocarcinoma | Carcinomas | Digestive |
| TGCT | Testicular germ cell tumors | Carcinomas | Genitourinary |
| THCA | Thyroid carcinoma | Carcinomas | Endocrine |
| THYM | Thymoma | Lymphoma | Respiratory |
| UCEC | Uterine corpus endometrial carcinoma | Carcinomas | Gynecology |
| UCS | Uterine carcinosarcoma | Mixed type | Gynecology |
| UVM | Uveal melanoma | Carcinomas | Eye |

Table S2: Correlation between MXD3 expression and methylation in various cancer types.

| cancertype | symbol | spm | fdr | entrez |
| --- | --- | --- | --- | --- |
| ACC | MXD3 | -0.26183 | 0.020009 | 83463 |
| BLCA | MXD3 | -0.18192 | 0.000226 | 83463 |
| BRCA | MXD3 | -0.19705 | 2.55E-07 | 83463 |
| CESC | MXD3 | -0.1648 | 0.003998 | 83463 |
| CHOL | MXD3 | -0.14157 | 0.408697 | 83463 |
| COAD | MXD3 | -0.26478 | 1.12E-05 | 83463 |
| DLBC | MXD3 | -0.17759 | 0.226453 | 83463 |
| ESCA | MXD3 | -0.23219 | 0.001555 | 83463 |
| GBM | MXD3 | -0.48027 | 0.000423 | 83463 |
| HNSC | MXD3 | -0.26172 | 1.57E-09 | 83463 |
| KICH | MXD3 | -0.45233 | 0.000161 | 83463 |
| KIRC | MXD3 | -0.37792 | 4.32E-12 | 83463 |
| KIRP | MXD3 | -0.18497 | 0.002141 | 83463 |
| LAML | MXD3 | -0.19847 | 0.009571 | 83463 |
| LGG | MXD3 | -0.33676 | 5.01E-15 | 83463 |
| LIHC | MXD3 | -0.28098 | 4.23E-08 | 83463 |
| LUAD | MXD3 | -0.23369 | 5.14E-07 | 83463 |
| LUSC | MXD3 | -0.2473 | 1.53E-06 | 83463 |
| MESO | MXD3 | -0.32239 | 0.002432 | 83463 |
| OV | MXD3 | -0.43333 | 0.249917 | 83463 |
| PAAD | MXD3 | -0.28249 | 0.000142 | 83463 |
| PCPG | MXD3 | -0.10681 | 0.154573 | 83463 |
| PRAD | MXD3 | -0.3784 | 0.6E-05 | 83463 |
| READ | MXD3 | -0.30933 | 0.002807 | 83463 |
| SARC | MXD3 | -0.25223 | 4.26E-05 | 83463 |
| SKCM | MXD3 | -0.21064 | 4.42E-06 | 83463 |
| STAD | MXD3 | -0.33182 | 6.78E-11 | 83463 |
| TGCT | MXD3 | -0.33627 | 2.88E-05 | 83463 |
| THCA | MXD3 | -0.23969 | 5.46E-08 | 83463 |
| THYM | MXD3 | -0.38558 | 1.6E-05 | 83463 |
| UCEC | MXD3 | -0.17473 | 0.021984 | 83463 |
| UCS | MXD3 | -0.10196 | 0.44939 | 83463 |
| UVM | MXD3 | -0.22869 | 0.041519 | 83463 |
